# Persistent Intraoperative Shock and Acute Kidney Injury After Liver Transplantation

**DOI:** 10.3390/jcm15114010

**Published:** 2026-05-22

**Authors:** Susana González-Suárez, Laura Llinares Espí, Manuel Grande Fernández, Juan José Ciudad Morales, Arantxa Vaque Cabeza, Clemente Antonio Durán Feliu, Paloma María Pereira Ricart, Lluís Castells Fuste, Gonzalo Sapisochin Cantis

**Affiliations:** 1 Department of Surgery, Unitat Docent Vall d’Hebron, Universitat Autònoma de Barcelona, Pg. de la Vall d’Hebron, 119-129, 08035 Barcelona, Spain; 2 Department of Anesthesiology, Hospital Universitari Vall d’Hebron, Pg. de la Vall d’Hebron, 119-129, 08035 Barcelona, Spain; 3 Cardiovascular Diseases Research Group, Vall d’Hebron Institut de Recerca (VHIR), Pg. de la Vall d’Hebron, 119-129, 08035 Barcelona, Spain; 4 Department of Internal Medicine—Liver Unit, Hospital Universitari Vall d’Hebron, Pg. de la Vall d’Hebron, 119-129, 08035 Barcelona, Spain; 5 Liver Diseases Research Group, Vall d’Hebron Institut de Recerca (VHIR), Pg. de la Vall d’Hebron, 119-129, 08035 Barcelona, Spain; 6 Department of Medicine, Unitat Docent Vall d’Hebron, Universitat Autònoma de Barcelona, Pg. de la Vall d’Hebron, 119-129, 08035 Barcelona, Spain; 7 CIBEREHD, Biomedical Research Networking Center for Hepatic and Digestive Diseases, Instituto de Salud Carlos III (ISCIII), 28029 Madrid, Spain; 8 Department of General, Abdominal Surgery & Liver Transplantation, Hospital Universitari Vall d’Hebron, Pg. de la Vall d’Hebron, 119-129, 08035 Barcelona, Spain; 9 CIRCATH Group, General, Digestive, Thoracic Surgery and Liver Transplantation, Vall d’Hebron Institut de Recerca (VHIR), Pg. de la Vall d’Hebron, 119-129, 08035 Barcelona, Spain

**Keywords:** liver transplantation, acute kidney injury, intraoperative shock, shock persistence, hemodynamic phenotype

## Abstract

**Background/Objectives**: Acute kidney injury (AKI) is a common complication after liver transplantation. Although intraoperative hypotension has been associated with its development, the impact of shock persistence and its hemodynamic profile remains poorly defined. **Methods**: This was a single-center retrospective observational study including 226 adult patients undergoing liver transplantation. Intraoperative shock was defined as a mean arterial pressure < 60 mmHg or a ≥30% decrease from baseline and was classified as hypovolemic, distributive, cardiogenic, or mixed based on pulmonary artery catheter data. AKI was defined according to Kidney Disease: Improving Global Outcomes (KDIGO) criteria within the first 7 postoperative days. Associations were assessed using adjusted logistic regression models. **Results**: Intraoperative shock occurred in 35.8% of patients, and the incidence of AKI was 52.2%. The presence of shock was not independently associated with AKI (adjusted OR 1.66; 95% CI 0.94–2.95). However, shock occurring in multiple phases of the procedure was associated with a higher incidence of AKI (81.8% vs. 50%; *p* = 0.010), greater severity, and higher mortality (27.3% vs. 3.4%; *p* = 0.002). In exploratory analyses, mixed shock was associated with an increased need for renal replacement therapy within 30 days (*p* = 0.006), persistent renal dysfunction at day 30 (*p* = 0.048), and higher mortality (*p* = 0.01), while hypovolemic shock was associated with moderate AKI (OR 6.60; *p* = 0.011). **Conclusions**: The presence of intraoperative shock alone is not independently associated with AKI. In contrast, its persistence is strongly associated with AKI development and worse clinical outcomes.

## 1. Introduction

Liver transplantation is a complex surgical procedure associated with marked intraoperative hemodynamic instability [1]. The pathophysiological alterations inherent to end-stage liver disease, together with the different phases of the procedure, lead to dynamic changes in preload, afterload, and cardiac output [1,2], often requiring advanced hemodynamic monitoring and support with vasopressors, inotropes, and transfusions to maintain adequate systemic perfusion [1,3].

Acute kidney injury is one of the most common complications following liver transplantation, with a reported incidence ranging from 30% to 60% [4,5]. It is associated with prolonged hospital stay, increased need for renal replacement therapy, and reduced survival [4,5,6,7]. Its pathophysiology is multifactorial and involves preoperative, intraoperative, and postoperative factors, including the severity of liver disease, ischemia–reperfusion injury, bleeding, impaired renal perfusion during surgery, and the nephrotoxic effects of calcineurin inhibitors used for immunosuppression [6,8,9,10].

In this context, intraoperative hypotension has been identified as an important determinant of AKI [11,12,13]. However, most available evidence has evaluated blood pressure as a global marker of hypoperfusion, without accounting for the underlying hemodynamic mechanism or the temporal dimension of hemodynamic instability [11,12,13,14].

The concept of intraoperative shock integrates both the magnitude of hypotension and its underlying pathophysiology, including states of hypovolemia, vasoplegia, and cardiac dysfunction [1,15,16]. However, the differential impact of these shock phenotypes on renal function after liver transplantation has not been fully characterized. Furthermore, the persistence of hemodynamic instability across the different phases of the procedure may play a key role in the development of renal injury, beyond isolated episodes, as suggested by recent studies on the duration of intraoperative hypotension [11,12].

In this context, the aim of the present study was to evaluate the association between the persistence and hemodynamic profile of intraoperative shock and renal outcomes after liver transplantation, analyzing their impact on the incidence, severity, and recovery of AKI, as well as on other relevant clinical outcomes.

Objectives

Primary objective

To evaluate the association between the persistence of intraoperative shock and the development of AKI within the first 7 days after liver transplantation.

Secondary objectives

- To explore the association between different hemodynamic profiles of intraoperative shock and renal outcomes, including AKI incidence and severity.
- To evaluate the relationship between the persistence of intraoperative shock and early AKI severity, including the development of moderate-to-severe AKI and the need for renal replacement therapy within 14 and 30 days.
- To assess the impact of different intraoperative shock profiles on renal outcomes, including renal recovery at day 7, and day 30, and the presence of persistent renal dysfunction at day 30.
- To evaluate the association between the persistence of intraoperative shock and clinical outcomes, including 30-day mortality, ICU length of stay, and hospital length of stay.

## 2. Materials and Methods

### 2.1. Study Design

This was a single-center retrospective observational study with prospective collection of intraoperative variables and retrospective review of medical records for preoperative and postoperative data. All patients provided informed consent for the use of their data for research purposes, and the study protocol was approved by the institutional Research Ethics Committee.

### 2.2. Study Population

Adult patients (≥18 years) who underwent liver transplantation between 12 January 2010, and 21 July 2022, were included. Only patients with intraoperative hemodynamic monitoring using a pulmonary artery catheter were considered.

Exclusion criteria included combined liver–kidney transplantation, preoperative renal replacement therapy or dialysis, portopulmonary syndrome, prior kidney transplantation, retransplantation during the same admission, missing intraoperative hemodynamic or postoperative renal function data, and death before postoperative day 2.

### 2.3. Anesthetic Management and Monitoring

General anesthesia was induced with fentanyl (2 μg/kg), propofol (2 mg/kg), and atracurium (0.5 mg/kg), and maintained with desflurane (3–8%), continuous fentanyl infusion (4 μg/kg/h), and atracurium (0.4 mg/kg/h).

Patients were ventilated in volume-controlled mode with a tidal volume of 8 mL/kg, positive end-expiratory pressure (PEEP) of 5 mmHg, and FiO_2_ of 0.55–0.60. Respiratory rate was adjusted to maintain an end-tidal CO_2_ (EtCO_2_) between 33 and 38 mmHg.

Monitoring included invasive arterial pressure and pulmonary artery catheter (Swan–Ganz). Intraoperative vasoactive support followed institutional protocols aimed at maintaining a mean arterial pressure (MAP) ≥ 60 mmHg. Norepinephrine infusion was primarily used for hemodynamic support, whereas epinephrine could be added in cases of insufficient response, particularly after reperfusion.

Transfusion and metabolic management were performed according to institutional protocols.

### 2.4. Definition of Intraoperative Shock and Hemodynamic Classification

Intraoperative shock was defined according to previously published definitions of intraoperative hypotension associated with postoperative organ dysfunction and AKI, as a MAP < 60 mmHg or a ≥30% decrease from baseline sustained for at least 10 min [17,18].

Shock episodes were classified according to the hemodynamic profile obtained from pulmonary artery catheter measurements as follows:

- Hypovolemic shock: low preload (central venous pressure (CVP) < 5 mmHg and/or pulmonary capillary wedge pressure (PCWP) < 8 mmHg), cardiac index (CI) < 2.2 L/min/m^2^, and normal or elevated systemic vascular resistance index (SVRI).
- Distributive shock: low SVRI (<1600 dyn·s·cm^−5^·m^2^) with preserved or elevated CI (≥2.5 L/min/m^2^).
- Cardiogenic shock: CI < 2.2 L/min/m^2^ with elevated filling PCWP (≥15–18 mmHg) or CVP (≥12 mmHg).
- Mixed shock: coexistence of two or more hemodynamic patterns.

### 2.5. Definition of Shock Persistence

Shock persistence was defined according to its distribution across the surgical phases of liver transplantation (hepatectomy, anhepatic phase, and reperfusion phase), and categorized as no shock, shock limited to a single phase, or shock present in multiple phases (≥2 phases). The analysis was based on the presence or absence of shock within each surgical phase rather than on cumulative duration or area-under-the-curve measurements of hypotension. This variable was considered the main measure of the temporal burden of hemodynamic instability.

### 2.6. Definition of Acute Kidney Injury

AKI was defined according to KDIGO criteria based on changes in serum creatinine within the first 7 postoperative days:

- Stage 1: 1.5–1.9-fold increase from baseline or ≥0.3 mg/dL.
- Stage 2: 2.0–2.9-fold increase.
- Stage 3: ≥3-fold increase, creatinine ≥ 4.0 mg/dL, or need for renal replacement therapy.

### 2.7. Data Collection and Outcome Definitions

Preoperative variables included age, sex, etiology of cirrhosis, hepatocellular carcinoma, comorbidities, Model for End-Stage Liver Disease (MELD) score at transplantation and Child–Pugh score, serum creatinine, estimated glomerular filtration rate, and portal vein thrombosis.

Intraoperative variables included hemodynamic parameters (MAP, CI, CVP, SVRI, PCWP), type of shock, use of vasopressors/inotropes, transfusions, cold ischemia time, and surgical duration.

Postoperative variables included serial serum creatinine measurements collected during the first 14 postoperative days. AKI was defined and staged according to KDIGO criteria using creatinine values from the first 7 postoperative days, which constituted the primary outcome of the study. Additionally, serum creatinine at postoperative day 30 was recorded to assess longer-term renal outcomes. Clinical outcomes included the need for renal replacement therapy within 14 and 30 days after transplantation, length of ICU and hospital stay, and 30-day mortality. Persistent renal dysfunction was defined as a serum creatinine ≥ 2 times baseline at day 30. Renal recovery at day 7 and day 30 was defined as the absence of KDIGO criteria for AKI and no requirement for renal replacement therapy.

### 2.8. Statistical Analysis

Categorical variables were expressed as frequencies and percentages and compared using the χ^2^ test or Fisher’s exact test. Continuous variables were expressed as mean ± standard deviation or median (interquartile range), depending on distribution, and compared using parametric or nonparametric tests.

The association between intraoperative shock persistence and the development of AKI was evaluated using a multivariable logistic regression model adjusted for clinically relevant variables (age, MELD score, baseline glomerular filtration rate, duration of the anhepatic phase, and cold ischemia time).

The association between shock persistence and in-hospital or 30-day mortality was also evaluated using an adjusted logistic regression model.

Results were reported as odds ratios (ORs) with 95% confidence intervals. A *p*-value < 0.05 was considered statistically significant.

All analyses were performed using IBM SPSS Statistics version 30.0 (IBM Corp., Armonk, NY, USA).

## 3. Results

### 3.1. Cohort Characteristics

A total of 226 patients undergoing liver transplantation during the study period were included. The mean age was 55.9 ± 10.5 years, and 26.1% were women. Baseline characteristics of the cohort are shown in Table 1.

Intraoperative shock occurred in 35.8% of patients (*n* = 81). The distribution of hemodynamic profiles was as follows: distributive shock in 19.9% (*n* = 45), mixed shock in 11.0% (*n* = 25), hypovolemic shock in 3.9% (*n* = 9), and cardiogenic shock in 0.8% (*n* = 2). Due to the small number of cases, cardiogenic shock was excluded from subgroup comparative analyses.

The overall incidence of AKI within the first 7 postoperative days was 52.2% (*n* = 118), including 24.3% (*n* = 55) stage 1, 18.6% (*n* = 42) stage 2, and 9.3% (*n* = 21) stage 3. Intraoperative variables according to the hemodynamic shock profile are presented in Table 2.

### 3.2. Intraoperative Shock Persistence and AKI

Intraoperative shock persistence was significantly associated with the development of AKI. The incidence of AKI of any grade was higher in patients with shock occurring in multiple phases of the procedure compared with those with shock limited to a single phase and those without shock (*p* = 0.01). Likewise, the incidence of severe AKI was higher in patients with involvement of multiple phases (two phases: 22.2% [*n* = 4]; three phases: 25.0% [*n* = 1]) compared with those with shock limited to a single phase (22.2% vs. 3.4% [*n* = 2]; *p* = 0.02).

**Figure 1:**
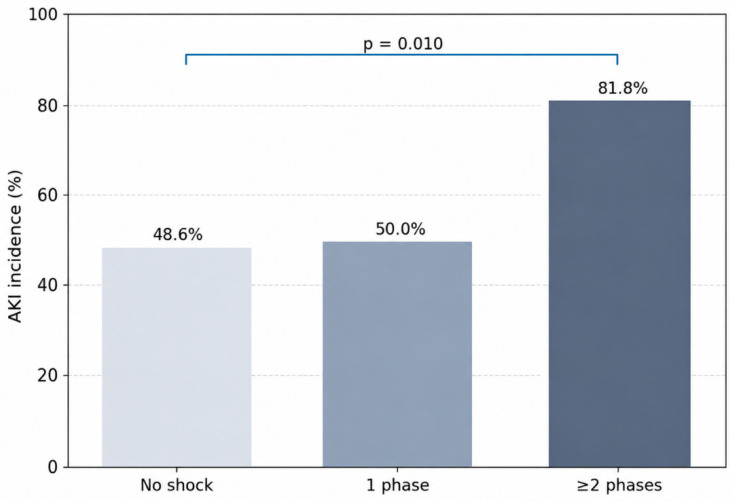
Incidence of acute kidney injury (AKI) according to the number of intraoperative shock phases.

The incidence of AKI according to the number of intraoperative shock phases is shown in Figure 1.

In the multivariable analysis, intraoperative shock persistence was independently associated with the development of AKI after adjustment for clinically relevant variables, including diabetes mellitus. Compared with patients without shock, the presence of shock in ≥2 phases was associated with a significantly increased risk of AKI (OR 4.49; 95% CI 1.42–14.22; *p* = 0.01), whereas shock limited to a single phase was not significantly associated with AKI (OR 1.15; 95% CI 0.62–2.16; *p* = 0.65). The results of the multivariable models are presented in Table 3.

**Figure 2:**
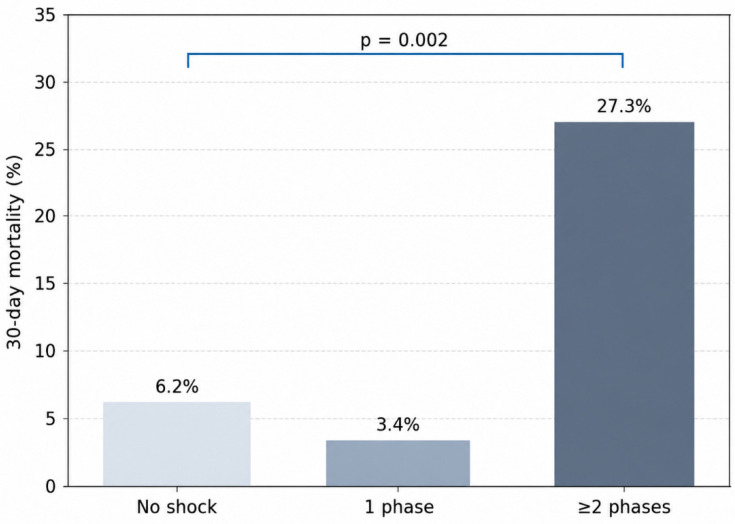
Thirty-day mortality according to the number of intraoperative shock phases.

Regarding clinical outcomes, 30-day mortality was higher in patients with shock occurring in multiple phases than in those with shock limited to a single phase (*p* = 0.002). Thirty-day mortality according to the number of intraoperative shock phases is shown in Figure 2.

### 3.3. Presence of Intraoperative Shock and AKI

As a complementary analysis, the association between the presence of intraoperative shock and the development of AKI was evaluated. In the bivariate analysis, the proportion of AKI was higher in patients with intraoperative shock compared with those without shock (59.3% [*n* = 48] vs. 48.3% [*n* = 70]), although the difference did not reach statistical significance (*p* = 0.11).

In the adjusted multivariable logistic regression analysis, the presence of intraoperative shock was not independently associated with the development of AKI (adjusted OR 1.66; 95% CI 0.94–2.95; *p* = 0.08) (Table 3).

### 3.4. Hemodynamic Profile of Shock and AKI

In an exploratory analysis, the association between different hemodynamic profiles of intraoperative shock and renal outcomes was evaluated. The incidence of AKI was higher in patients with mixed shock (72% [*n* = 18]), followed by hypovolemic shock (66.7% [*n* = 6]) and distributive shock (48.9% [*n* = 22]), although no statistically significant differences were observed in the overall analysis.

In the adjusted multivariable analysis, no significant independent associations were found between shock profiles and the development of AKI.

**Figure 3:**
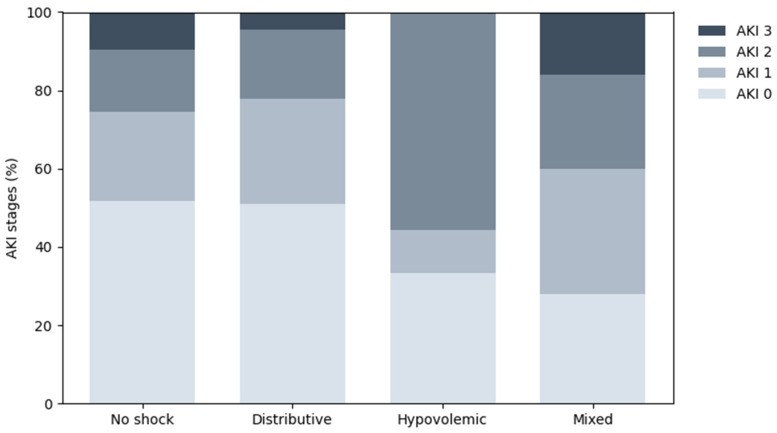
Distribution of AKI stages (KDIGO) according to type of intraoperative shock.

Regarding AKI severity, no significant differences were observed across shock types overall. However, in the subgroup analysis according to KDIGO stages, hypovolemic shock was associated with a higher risk of moderate AKI (OR 6.60; *p* = 0.011). The distribution of AKI stages according to different shock profiles is shown in Figure 3.

### 3.5. Renal Outcomes According to Shock Type

The incidence of renal replacement therapy was higher in patients with mixed shock both within the first 14 postoperative days and at 30 days (23.5% [4 of 17] in both cases), whereas no patients with hypovolemic shock required renal replacement therapy (0% [0/5]) (*p* = 0.002 and *p* = 0.006, respectively).

Among patients who developed AKI, renal recovery was observed in 64.4% (*n* = 76) at day 7 and increased to 74.6% (*n* = 88) by day 30, without significant differences between groups, including patients without shock and those with different shock phenotypes (*p* = 0.39 and *p* = 0.27, respectively). Persistent renal dysfunction occurred in 11.0% (*n* = 13) of patients and was more frequent in patients with mixed shock (35.3%, *n* = 6; *p* = 0.048).

### 3.6. Clinical Outcomes According to Shock Type

In the analysis according to hemodynamic profile, 30-day mortality was significantly higher in patients with mixed shock (23.5% [*n* = 6]) compared with those with distributive (2.2% [*n* = 1]) or hypovolemic shock (0% [*n* = 0]) (*p* = 0.01).

No significant differences were observed in ICU or hospital length of stay according to shock type.

## 4. Discussion

In this observational study of patients undergoing liver transplantation, a high incidence of AKI was observed in the early postoperative period, consistent with previous reports in the literature [4,5,6,7]. The main finding is that the isolated presence of intraoperative shock was not independently associated with the development of AKI after adjustment for relevant clinical factors. In contrast, both the hemodynamic profile of shock and its persistence across the different phases of the procedure were associated with worse clinical outcomes.

Furthermore, our results indicate that not all shock phenotypes have the same clinical impact. While hypovolemic shock was associated with an increased risk of moderate AKI, mixed shock was consistently associated with the worst clinical outcomes, including a higher need for renal replacement therapy, a higher frequency of persistent renal dysfunction, and increased mortality. Notably, the absence of renal replacement therapy requirement in patients with hypovolemic shock, despite its association with moderate AKI, suggests that not all patterns of renal injury have the same propensity to progress to more severe forms. These findings suggest that the hemodynamic profile of shock represents a relevant determinant of renal outcomes beyond the mere presence of hemodynamic instability. In particular, mixed shock may reflect a more severe pathophysiological condition, in which the coexistence of hypovolemia and systemic vasodilation more profoundly compromises effective renal perfusion, as suggested by previous studies on the interaction of hemodynamic mechanisms [19,20,21].

Regarding temporal dynamics, involvement of multiple phases of the procedure was consistently associated with a higher incidence and severity of AKI, as well as increased mortality, suggesting that the temporal burden of hemodynamic instability is a key determinant of renal injury. Consistently, patients with shock limited to a single phase of the procedure showed a clinical course similar to those without shock, suggesting the existence of a threshold of tolerance to hemodynamic instability. This finding reinforces the concept that renal risk depends not only on the occurrence of hypotensive episodes, but also on their cumulative burden and the physiological context in which they occur, in line with studies highlighting the role of cumulative exposure to hemodynamic instability [11,12].

These results add an important nuance to previous evidence that has identified intraoperative hypotension as a risk factor for AKI [11,12,13]. Most studies have focused on arterial pressure as a global marker of hypoperfusion, without considering its cumulative duration or the underlying physiological context [11,12,13]. Our findings reinforce that both the duration and the physiological context of hemodynamic instability are key determinants of its clinical impact, and that brief or phase-limited exposures may be well tolerated, whereas persistent or repeated episodes are associated with a higher risk of renal injury and worse clinical outcomes, consistent with previous studies emphasizing the importance of the duration of intraoperative hypotension [11,12].

From a pathophysiological perspective, the association between persistent shock and AKI is consistent with mechanisms described in liver transplantation, where renal hypoperfusion, systemic inflammation, and impaired renal autoregulation play a key role [6,8,9,10]. During the procedure, particularly in the reperfusion phase, abrupt hemodynamic changes may compromise renal perfusion [22,23,24]. In this context, repeated or prolonged episodes of hemodynamic instability may promote the development of acute tubular injury and explain the higher incidence and severity of AKI observed in patients with persistent shock [6,25]. Diabetes mellitus was also associated with postoperative AKI in the adjusted analysis, suggesting that pre-existing metabolic and microvascular alterations may increase renal vulnerability during liver transplantation. Additionally, the trend toward higher requirements for platelet and fresh frozen plasma transfusion in patients with intraoperative shock may reflect greater bleeding severity or coagulation disturbances, which could further contribute to renal hypoperfusion and AKI development.

Despite the high incidence of AKI, renal function recovery was frequent, suggesting that a substantial proportion of cases may correspond to reversible forms of renal dysfunction, likely related to transient hemodynamic alterations.

In contrast, distributive shock was not clearly associated with worse renal outcomes. This finding may be interpreted in the context of the hyperdynamic circulatory state characteristic of advanced liver disease, in which reduced systemic vascular resistance is part of the baseline physiology [26,27,28]. In this setting, the intraoperative distributive pattern may represent an exacerbation of a pre-existing condition, with a comparatively lower impact on renal perfusion than other shock phenotypes [28,29,30,31].

Overall, these findings suggest that the impact of hemodynamic instability on renal function depends not only on the presence of hypotension, but also on the physiological context in which it occurs.

From a clinical perspective, these findings suggest that shock persisting across more than one surgical phase should prompt early reassessment of the underlying hemodynamic mechanism using advanced monitoring, optimization of preload and transfusion strategy, and timely adjustment of vasoactive or inotropic support according to the shock phenotype [19,32].

This study has several limitations. Its observational design, despite adjustment for clinically relevant variables, does not allow exclusion of residual confounding. In addition, some subgroups, particularly cardiogenic shock, included a limited number of patients, restricting the interpretation of subgroup analyses. Furthermore, potentially relevant intraoperative variables such as fluid balance, urine output, or exposure to nephrotoxic agents were not included in the adjusted analyses. Intraoperative lactate dynamics were also not analyzed. The study evaluated phase-based shock persistence rather than continuous hemodynamic exposure metrics such as cumulative hypotension duration or hypotension area-under-the-curve. Finally, this is a single-center study, which may limit the generalizability of the findings.

## 5. Conclusions

In patients undergoing liver transplantation, the isolated presence of intraoperative shock is not independently associated with the development of AKI. However, its persistence across multiple phases of the procedure and its hemodynamic profile, particularly mixed shock, are associated with worse renal outcomes and increased mortality. These findings highlight the relevance of both the temporal dynamics and the underlying mechanism of hemodynamic instability as key determinants of renal risk in this setting.

## Figures and Tables

**Table 1:** Baseline characteristics according to intraoperative shock.

	Total (*n* = 226)	No Shock (*n* = 145)	Shock (*n* = 81)	*p*-Value
Sex				0.71
Female	59 (26.1)	39 (26.9)	20 (24.7)	
Male	167 (73.9)	106 (73.1)	61 (75.3)	
Alcohol-related cirrhosis	108 (48.2)	68 (46.9)	40 (49.4)	0.72
NASH	15 (6.3)	9 (6.2)	6 (7.4)	0.72
Fulminant liver failure	4 (1.8)	3 (2.1)	1 (1.2)	0.64
Autoimmune liver disease	9 (4.0)	6 (4.1)	3 (3.7)	0.87
Primary biliary cholangitis	15 (6.7)	7 (4.8)	8 (9.9)	0.14
Viral cirrhosis	88 (39.3)	59 (40.7)	30 (37.0)	0.55
Other etiologies	21 (9.3)	15 (10.3)	6 (7.4)	0.46
Diabetes mellitus	65 (28.8)	48 (33.1)	17 (21.0)	0.054
Hypertension	62 (27.4)	45 (31.0)	17 (21.0)	0.10
Heart disease	17 (7.5)	13 (9.0)	4 (4.9)	0.27
Portal thrombosis	34 (15.0)	23 (15.9)	11 (13.6)	0.64
Child–Pugh class				0.51
A	64 (28.3)	42 (29.0)	22 (27.2)	
B	78 (34.5)	53 (36.6)	25 (30.9)	
C	84 (37.2)	50 (34.5)	34 (42.0)	
Hepatocellular carcinoma	105 (46.5)	72 (49.7)	33 (40.7)	0.19
Creatinine at transplantation (mg/dL)	0.92 ± 0.55	0.90 ± 0.62	0.95 ± 0.42	0.20
eGFR at transplantation (mL/min/1.73 m^2^)	84.40 ± 20.38	84.33 ± 18.73	85.53 ± 23.14	0.69
MELD score	19.38 ± 6.08	19.19 ± 5.82	19.70 ± 6.55	0.80

Data are presented as *n* (%) or mean ± standard deviation. Categorical variables were compared using the χ^2^ test. Continuous variables were compared using the Kruskal–Wallis test. Abbreviations: eGFR, estimated glomerular filtration rate; MELD, Model for End-Stage Liver Disease; NASH, nonalcoholic steatohepatitis.

**Table 2:** Intraoperative variables according to shock status.

	Total (*n* = 226)	No Shock (*n* = 145)	Shock (*n* = 81)	*p*-Value
Norepinephrine dose (mg)	8.0 (3.0–14.4)	8.0 (3.0–14.0)	9.0 (4.0–15.0)	*p* = 0.35
Adrenaline dose (mg)	0.0 (0.0–0.05)	0.0 (0.0–0.01)	0.0 (0.0–0.2)	*p* = 0.007
Calcium administration (g)	2.5 (1.0–5.0)	2.0 (1.0–4.0)	3.0 (1.5–5.0)	*p* = 0.16
PRBC (units)	3.0 (1.0–6.0)	3.0 (1.0–6.0)	4.0 (2.0–6.0)	*p* = 0.10
FFP (units)	4.0 (0.0–6.0)	3.0 (0.0–6.0)	4.0 (0.0–8.0)	*p* = 0.08
Platelets (units)	0.0 (0.0–2.0)	0.0 (0.0–1.0)	1.0 (0.0–2.0)	*p* = 0.06
Hepatectomy phase (min)	159.0 (145.0–185.0)	160.0 (142.0–190.0)	150.0 (145.0–180.0)	*p* = 0.46
Anhepatic phase (min)	53.0 (45.0–67.0)	55.0 (45.0–70.0)	50.0 (45.0–60.0)	*p* = 0.38
Cold ischemia (min)	334.5 (300.0–385.0)	340.0 (300.0–386.0)	330.0 (300.0–380.0)	*p* = 0.54
Surgery duration (min)	480.0 (450.0–540.0)	480.0 (450.0–540.0)	480.0 (450.0–540.0)	*p* = 0.65
Bicarbonate (mEq)	200.0 (100.0–250.0)	170.0 (125.0–250.0)	200.0 (100.0–290.0)	*p* = 0.15

Data are presented as median (interquartile range). Continuous variables were compared using the Kruskal–Wallis test. Vasopressor doses are presented as descriptive intraoperative management variables. Abbreviations: FFP, fresh frozen plasma; PRBC, packed red blood cells.

**Table 3:** Multivariable logistic regression models for acute kidney injury (AKI).

	Model 1: Any Shock OR (95% CI)	*p*-Value	Model 2: Shock Persistence Across Surgical Phases OR (95% CI)	*p*-Value
Shock	1.66 (0.94–2.95)	0.08		
1 phase			1.15 (0.62–2.16)	0.65
≥2 phases			4.49 (1.42–14.22)	0.01 *
MELD score	1.02 (0.97–1.07)	0.47	1.01 (0.97–1.06)	0.55
Preoperative GFR	1.01 (1.00–1.02)	0.21	1.01 (1.00–1.02)	0.23
Anhepatic phase (min)	1.01 (1.00–1.01)	0.16	1.00 (1.00–1.01)	0.28
Cold ischemia time (min)	1.00 (1.00–1.01)	0.09	1.00 (1.00–1.01)	0.13
Diabetes mellitus	1.92 (1.03–3.59)	0.04 *	1.86 (0.99–3.50)	0.05

Data are presented as odds ratios (ORs) with 95% confidence intervals (CIs). Associations were evaluated using adjusted multivariable logistic regression models. Abbreviations: CI, confidence interval; GFR, glomerular filtration rate; MELD, Model for End-Stage Liver Disease; OR, odds ratio; *, *p* < 0.05.

## Data Availability

Data are contained within the article. The original contributions presented in this study are included in the article.

## Abbreviations

The following abbreviations are used in this manuscript:

AKI	Acute Kidney Injury
CI	Cardiac Index
CVP	Central Venous Pressure
KDIGO	Kidney Disease: Improving Global Outcomes
MAP	Mean Arterial Pressure
MELD	Model for End Stage Liver Disease
OR	Odds ratio
PCWP	Pulmonary Capillary Wedge Pressure
SVRI	Systemic Vascular Resistance Index
